# Digital and Community-Engaged Research Methods to Adapt Savvy Caregiver® for LGBTQIA+ Caregivers

**DOI:** 10.1093/geroni/igaf122.551

**Published:** 2025-12-31

**Authors:** Joel Anderson, Jace Flatt, Joseph Knight, Brittany Klenczar-Castro, John Hobday, Carey Sherman

**Affiliations:** University of Tennessee, Knoxville, Tennessee, United States; University of Las Vegas, Nevada, United States; University of Tennessee, Knoxville, Tennessee, United States; University of Las Vegas, Nevada, United States; Savvy Systems LLC, Minneapolis, Minnesota, United States; Savvy Systems LLC, Minneapolis, Minnesota, United States

## Abstract

While caring for people with dementia affects all caregivers, LGBTQIA+ caregivers experience unique minority stressors, including discrimination and stigma, that magnify the strains of caregiving. Our research and that of others finds LGBTQIA+ caregivers have significantly poorer health than heterosexual, cisgender (not transgender) caregivers, and experience higher levels of physical and emotional stress and caregiver burden. These higher levels of stress may be related to LGBTQIA+ caregivers having less access to social and community supports. The Savvy Caregiver® program is a successful psychoeducation intervention that has been shown to relieve dementia caregivers’ distress and depressive symptoms as well as enhance caregivers’ confidence, knowledge, and behavioral skills, outcomes of specific importance to LGBTQIA+ caregivers. Here, we present our combination of digital and community-engaged research methods and user-centered design approaches to develop a culturally adapted version of Savvy Caregiver®. This study is innovative and has potential for immediate impact as, to our knowledge, it will be the first cultural adaptation of Savvy Caregiver® for LGBTQIA+ caregivers of people with dementia.

